# Writing about a stressful experience can impair visual working memory

**DOI:** 10.1371/journal.pone.0304406

**Published:** 2024-07-05

**Authors:** Colton L. Hunter, Grant S. Shields

**Affiliations:** Department of Psychological Science University of Arkansas, Fayetteville, AR, United States of America; University of Jyväskylä: Jyvaskylan Yliopisto, FINLAND

## Abstract

Acute stress has been well-established to impair working memory. However, less is known about how writing about an unresolved stressor may influence working memory or working memory processes. We addressed these issues in the present study (*N* = 282) by randomly assigning participants to write about an unresolved stressful experience (stressful writing condition or the events of the previous day). We then both measured performance on a change detection task and used computational modeling to estimate the processes underlying performance: attention, capacity, and guessing bias. We found that, relative to the control condition, writing about a stressful experience impaired change detection task performance and significantly impaired task attention. These results show that the effects of writing about an unresolved stressor may mimic the effects of acute stress on working memory, rather than conforming to expectations from mood-as-information theory.

## Introduction

Acute stress impairs working memory—the small amount of information that can be held in mind and used in the execution of cognitive tasks [1, 2] This effect has been attributed to a shift in resource allocation towards processes relevant to the stressor at hand and way from process deemed irrelevant for immediate survival [2]. Although the effects of acute stress on working memory are well established, it is unclear whether reexperiencing a stressor mimics the cognitive effects of experiencing an acute stressor. Previous work has examined the effects of writing about a stressful experience on both working memory and long-term memory [3, 4] but obtained seemingly discrepant results, with sex differences possibly playing a role. This study addressed this issue by probing this dynamic when examining the effect of writing about a stressful experience on working memory.

Writing about an unresolved stressful experience involves aspects of memory and interpretations of prior stressful experiences, rather than current exposure. Other affect inductions, which induced negative affect using emotional stimuli, threat-of-shock, and test-based anxiety, have not been found to impact working memory in the same way as acute stress [5, 6]. Writing-based affect inductions are particularly effective affect inductions that may reinstate the psychological state from the recalled stressor. Consistent with this in a small number of ways, this manipulation has been found to induce a physiological response that is consistent with a mild stress induction or a strong negative affect induction, such as increased heart rate and blood pressure, greater proinflammatory cytokine activity, reduced affect, and impaired executive function [7–9]. Although this manipulation produces a similar subjective state, its effects on working memory are less clear.

One potentially important factor in determining the effects of writing about a stressful experience on working memory is sex. The effects of acute stressors are not identical across sexes. Stress has been found to differentially influence memory performance, emotion regulation, and working memory in men and women [2, 10]. Specifically, regarding working memory, exposure to an acute stressor enhances working memory in men while at the same time impairing it in women [10]. Additionally, there are also relatively clear-cut sex differences in the psychological benefits of writing about an unresolved stressful experience. Although it is beneficial across sexes, these emotional benefits tend to be stronger in men than in women [11, 12]. Coupled with sex differences in the effects of acute stress on working memory, these results suggest that should writing about a stressful experience closely mimic exposure to an acute stressor, there should be a similar interaction with participant sex such that men experience an enhancement in working memory whereas women experience an impairment.

Impairments in working memory following an acute stressor have been attributed to a shift in resource allocation [2]. Resources are diverted away from stressor-irrelevant processes and towards stressor-relevant processes as a means of dealing with the stressor. Consequently, certain aspects of working memory performance, such as attention, may be more preferentially inhibited than others, such as capacity and guessing bias, as attentional resources are pulled away from task-relevant stimuli and towards high-priority stress-relevant processes. Should the effects of writing about an unresolved stressor mimic experiencing an acute stressor, we should expect to see a similar shift in resource allocation towards the stressor, resulting in both an overall decrease in working memory performance and attention to the task, along with relatively little change in other working memory processes.

As mentioned previously, two recent studies have examined the effects of writing about an unresolved stressful experience on working memory and long-term memory, respectively [3, 4]. However, these studies obtained potentially discrepant results. The first study [4] found no significant effect of writing about a stressful experience of overall working memory or working memory components. The second study [3], however, found a significant effect of the writing manipulation on memory retrieval strategies in men but not women. Given the significant interaction between writing condition and sex observed in Hunter et al., we suspected that the null finding observed regarding working memory may be in part to an unexamined interaction between the writing condition and sex. With a sample size of only 171 participants, the previous study may have had insufficient power to detect such an effect, especially given how weak the effects of writing-based manipulations tend to be [7, 9]. Therefore, replication of the study conducted by Shields et al. with a larger sample size, in order to explore this potential sex by writing condition interaction.

### Current research

We addressed the question of whether there might be sex differences in the effects of writing about an unresolved stressful experience on working memory and its component processes by randomly assigning participants to write about either an unresolved stressful experience or the neutral events of the previous day. Shortly after the writing task, we assessed working memory via a change detection task and estimated component working memory processes by fitting a fixed capacity model of working memory [13] to task data. We hypothesized that participants in the stressful writing condition would have poorer overall working memory performance, and that this effect would be stronger in women than in men.

## Method

### Participants

An unpublished secondary analysis of data from the study we were attempting to replicate [4] using a 2x2 between-factor ANOVA predicting overall working memory performance from writing condition and sex found a sex by writing condition interaction in predicting working memory task performance at set size five, with an effect size of *f* = 0.16 (data available for that study on OSF) [4]. This analysis was conducted in R, version 4.2.2, using the car package. We sought to replicate this effect. Measures included in this study were added to a larger study as data collection was ongoing. As a result, data collection was stopped once the larger study met its power analysis target, resulting in a sample of 284 participants prior to exclusions, with two participants being excluded from analyses. This sample was approximately 1.6 times larger than the prior study conducted by Shields et al. and provided 76% power to detect the observed interaction. Data were collected from May 2^nd^ of 2021 to April 29^th^ of 2022. Participants received extra credit for participating. Participants were randomly assigned via Qualtrics to either the stressful writing condition (*n* = 128; 60.9% women) or control condition (*n* = 154; 55.8% women). Of this sample, 82.1% were White, 7.9% Hispanic or Latine, 5.7% African American, 3.1% Asian American, 1.8% Native American, and 1.2% Native Hawaiian or Pacific Islander. Age, sex, and race/ethnicity did not significantly differ between the stressful writing and control conditions, *p*s>.179.

### Materials

#### Essay manipulation’

Participants were given six minutes to type an essay. Participants in the stressful writing condition were given the following prompt:

“Please write an essay in the space provided below. Please remember, relive, and vividly recall a negative event that makes you feel extremely stressed out. Choose an event that has not been resolved and is still a source of stress for you. Please give as much detail as necessary to vividly describe the situation and why it stresses you out. You will have six minutes to complete this task. You must write for the full six minutes. The study will automatically continue when the six minutes is over as long as you have written something, but you will not be able to complete the study if you do not write anything.”

Participants in the control (i.e., neutral writing) condition were given the following prompt:

“Please write an essay in the space provided below. Please remember, relive, and vividly recall all of the events that happened to you yesterday. Please describe any and all events regardless of whether they were routine or unusual. Please give as much detail as necessary to vividly describe the situation. You will have six minutes to complete this task. You must write for the full six minutes. The study will automatically continue when the six minutes is over as long as you have written something, but you will not be able to complete the study if you do not write anything.”

These prompts and essay timings were identical to those used by Shields et al. [4].

#### Manipulation check

Immediately after the writing task, participants were asked two questions about the writing task they just completed: “How stressful was the writing task you just completed?” and, “How unpleasant was the writing task you just completed?” Participants responded using visual analogue scales bounded at 0 (“Not at all stressful/unpleasant”) and 100 (“Extremely stressful/unpleasant”). The scale was marked with numbers in increments of 10, but responses were not restricted to these numbers.

#### Writing sentiment

As an additional manipulation check, participants’ responses to the writing task were analyzed for sentiment (i.e., overall valence of participants’ essay based upon words used) using the sentimentr package, version 2.9.0, in R. Sentiment scores were calculated for each sentence within each participant’s essay based on word usage such that positive values indicated more positive sentiment and negative values indicated more negative sentiment. Scores for each sentence were then summed across sentences for each participant to produce an overall sentiment score for their essay. Additional sentiment scores were also created by averaging scores across sentences to produce an overall sentiment score. These results did not significantly differ from results obtained when summing scores, therefore subsequent analyses were conducted using summed scores.

#### Visual working memory

**Fig 1:**
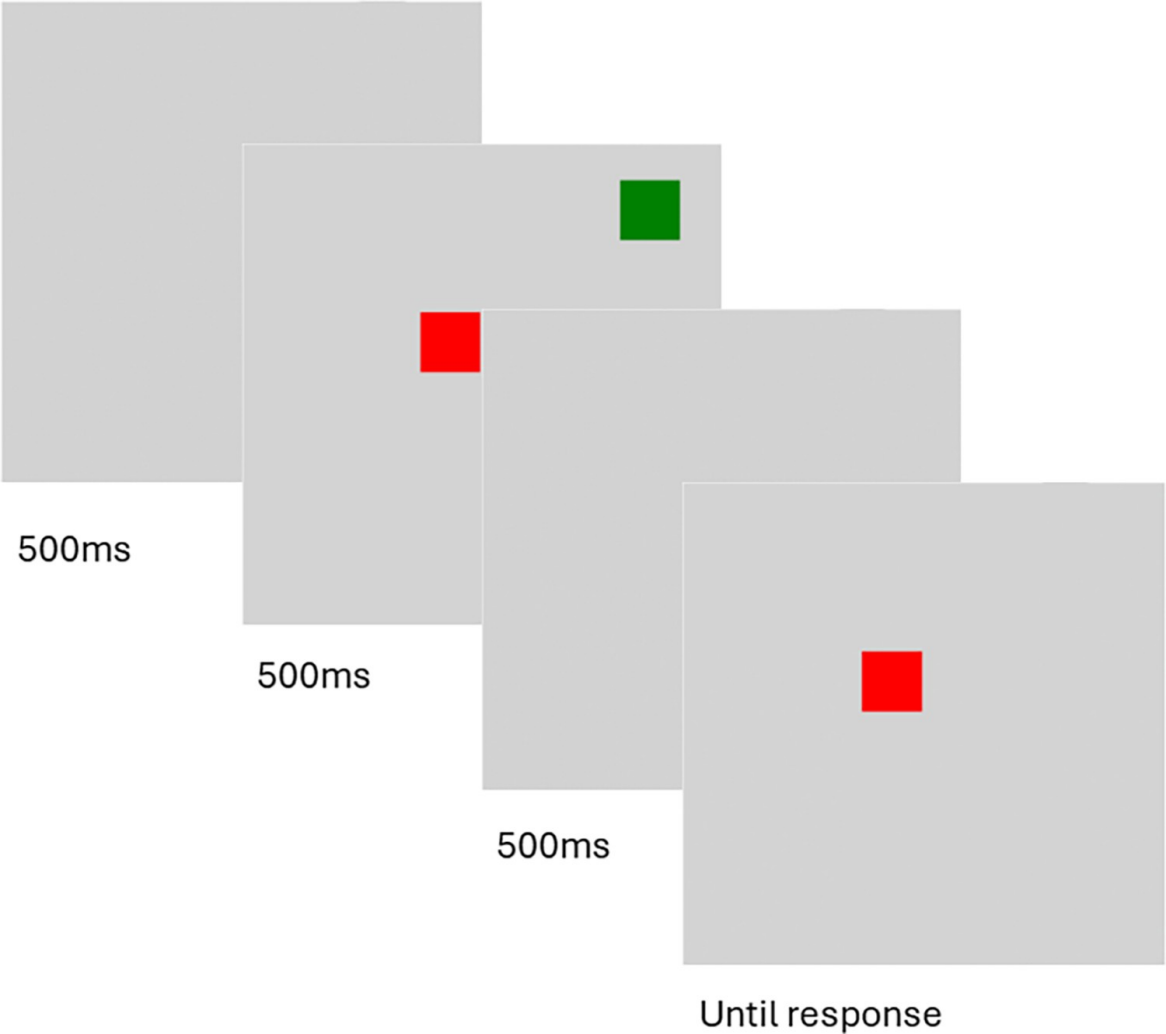
Change detection task used to assess visual working memory.

Working memory was measured using a change detection task adapted from Shields et al. [4], coded in Inquisit. The task consisted of 18 practice trials and 126 test trials divided into 3 blocks of 42 trials each. The break time between blocks was unlimited. Each trial began with a blank screen presented for 500ms, after which a non-overlapping randomly positioned colored array of 2, 5, or 8 squares was presented for 500ms. Colors were sampled from a pool of seven possible colors. RGB values for each color are default values used for the strings, “white”, “black”, “purple”, “green”, “red”, “blue”, and “yellow”. Colors were not repeated except in the case of set size eight, where one color of the seven was repeated randomly. The height and width of the squares were 11% of each participant’s monitor height. The square array was then replaced by a blank screen for 500ms—note that Shields et al. [4] masked squares rather than presented a blank screen, and this design difference, because masks suppress iconic memory whereas a blank screen does not [14], has previously been found to play an important role in change detection task effects [13]. Next, a single colored target square appeared and remained on the screen until participants indicated whether it was the same color it had been at study. The target square had a 50% chance of remaining the same color as when initially presented; if the target square had changed color, the new color was randomly sampled from the remaining colors. After a participant provided their response, feedback was given for 500ms (see Fig 1).

### Procedure

**Fig 2:**
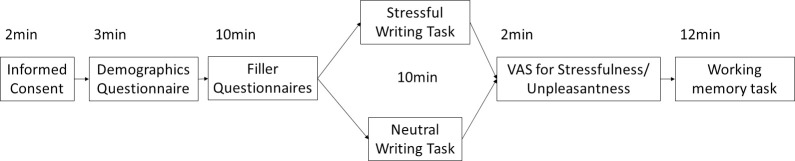
General procedures for the study.

Study procedures were completed online. Participants completed an informed consent form, providing written consent, followed by a demographic questionnaire. Participants then completed filler self-report questionnaires consisting of two self-esteem questionnaires, two questionnaires assessing chronic stress, and a personality questionnaire. This study was part of a larger study for which these questionnaires were important. Participants then completed either the stressful writing task, in which they wrote about an unresolved stressful experience, or the control writing task, in which they wrote about the events of the previous day, depending upon their assigned condition. Following the writing manipulation, participants were presented with the visual analogue scale assessing the stressfulness and unpleasantness of the writing task as a manipulation check. Finally, participants completed the change detection task (see Fig 2).

### Data analysis

Individual participant responses were examined for complying with study instructions prior to data analysis. Two participants were excluded from analyses because of this, with both participants writing something unrelated to the instructions of the writing task.

Bias-corrected change detection scores (i.e., *d*’) were calculated by subtracting false alarms from z-scored hits, both in total and at each set size, using the log-linear transformation given by Snodgrass and Corwin [15] to avoid infinite estimations. Working memory performance was further analyzed by fitting data to a fixed-capacity model of working memory, adapted from Rouder et al. [13], based on participant hits, false alarms, misses, and correct rejections at each set size (*i*):

$$d_{i}=\min\left(1,\frac{k}{{set\;size}_{i}}\right)$$

$${hits}_{i}=a(d_{i}+(1-d_{i})*g)+(1-a)*g$$

$${misses}_{i}=1-{hits}_{i}$$

$${false\;alarms}_{i}=a(1-d_{i})*g+(1-a)*g$$

$${correct\;rejections}_{i}=1-{false\;alarms}_{i}$$

Parameters for attention (*a*), capacity (*k*), and guessing bias (*g*) were estimated via maximum likelihood using the nmkb function in the dfoptim R package, version 2020.10–1. The probability that a probed item was in memory (*d*) was equal to *k*/set size if the set size exceeded capacity and equal to 1 if set size was not larger than capacity. Parameter constraints were such that capacity was constrained to be > = 1, attention to > = .7, and guessing bias to > = 0 and < = 1.

Type III sum of squares ANOVAs were run using the car package in R. Four ANOVAs were used to examine the effect of Condition (stressful writing; neutral writing)—and potential moderation by Sex (men; women)—on overall working memory performance, as well as the component estimated parameters of attention, capacity, and guessing bias. All data analyses were conducted using R, version 4.2.2. Syntax and data are available upon request.

### Transparency and openness

We report how we determined our sample size, all data exclusions, all manipulations, and all measures in the study. All data, analysis syntax, and research materials are available upon request. Data were analyzed using R, version 4.2.2, using the car package version 3.1–1, the dfoptim package version 2020.10–1, and the sentiment package version 2.9.0. This study’s design and its analyses were not pre-registered.

## Results

### Manipulation check

**Fig 3:**
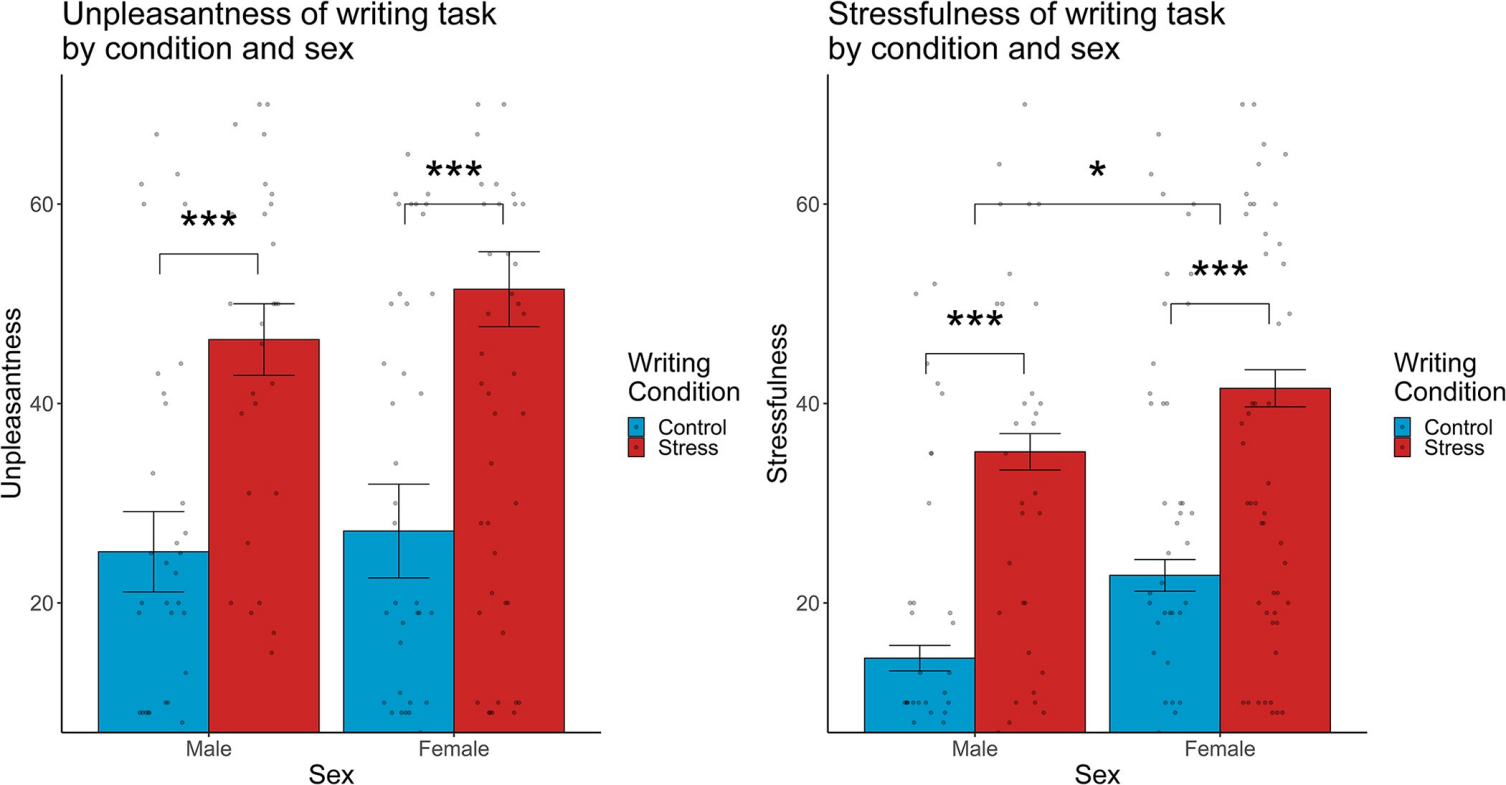
Mean unpleasantness (**A**) and stressfulness (**B**) of the writing task grouped by experimental condition and participant sex. Participants in the stressful writing condition rated the task as significantly more unpleasant and stressful than those in the neutral condition. Women rated either task as significantly more stressful than men. ***p < .001, *p < .05.

We first examined whether participants in the stressful writing condition rated the writing task as more stressful and unpleasant than participants in the neutral writing task. Two-tailed t tests were conducted to examine differences in self-reported stressfulness and unpleasantness between writing conditions. As expected, participants in the stressful writing condition (*M*_*Stress*_ = 39.08, *SE*_*Stress*_ = 2.76) rated the writing task as significantly more stressful than participants in the neutral writing condition (*M*_*Control*_ = 18.81, *SE*_*Control*_ = 2.03), *t*(275) = 6.01, *p* < .001, Cohen’s *d* = 0.72, 95% CI [-26.91, -13.63]. Similarly, participants in the stressful writing condition (*M*_*Stress*_ = 49.41, *SE*_*Stress*_ = 3.03) rated the writing task as significantly more unpleasant than participants in the neutral writing task (*M*_*Control*_ = 26.27, *SE*_*Control*_ = 2.59), *t*(275) = 5.84, *p* < .001, Cohen’s *d* = 0.70, 95% CI [-30.94, -15.35]. Additionally, we observed a sex difference in reported stressfulness of that task: Women (*M* = 31.47, *SE* = 2.38) reported significantly greater stressfulness than men (*M* = 23.46, *SE* = 2.64), *t*(275) = 2.22, *p* = .027, Cohen’s *d* = 0.27, 95% CI [-15.10, -0.92]. There was no significant sex difference observed for reported unpleasantness of the writing task, *t*(275) = 1.06, *p* = .290. Sex did not interact with condition to predict self-reported stressfulness or unpleasantness of the writing task, *p*s>.707 (see Fig 3).

As an additional manipulation check, essay sentiment was examined between writing conditions. Participants’ essays in the stressful writing condition (*M*_*Stress*_ = -0.03, *SE*_*Stress*_ = 0.05) overall had significantly more negative sentiment than participants’ essays in the neutral writing condition (*M*_*Control*_ = 0.83, *SE*_*Control*_ = 0.05), *t*(275) = 12.04, *p* < .001, Cohen’s *d* = 1.45, 95% CI [0.72, 0.99].

### Primary analyses

**Fig 4:**
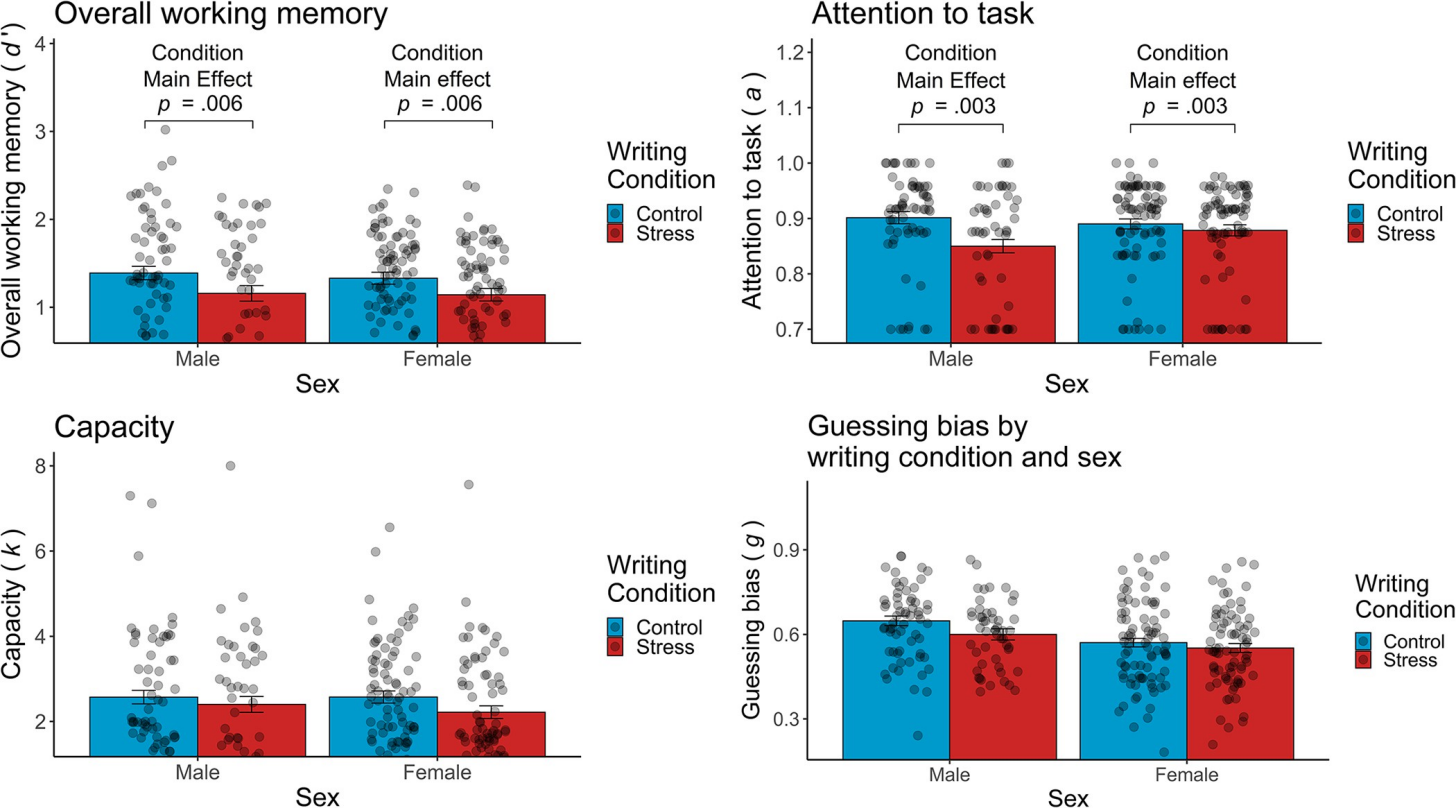
Overall working memory and working memory parameters by writing condition and participant sex. Overall working memory and attention to the task were significantly lower in the stressful writing condition. No significant effect of condition or sex was observed on capacity. Men showed a greater guessing bias in guessing “old” than women. ***p < .001, **p < .01, *p < .05.

For our primary analyses we first examined whether overall working memory performance differed as a function of writing condition and sex. In a 2x2 ANOVA predicting overall working memory performance from Condition (stressful writing, neutral writing) and Sex (men, women) a main effect of Condition emerged, *F*(1, 273) = 7.74, *p* = .006, whereas neither the predictor Sex nor the Condition×Sex interaction reached significance, *p*s>.421. Exploring the main effect of Condition, we found that participants in the stressful writing condition (*M* = 1.16, *SE* = 0.056) had significantly worse overall performance on the change detection task than participants in the neutral writing condition (*M* = 1.37, *SE* = 0.051) (see Fig 4). In other words, contrary to prior work, we found that writing about a stressful event impaired working memory performance broadly.

As an additional analysis, we examined whether overall working memory performance differed as a function of set size, writing condition, and sex. These results are summarized in Table 1.

**Table 1:** Overall working memory performance as a function of set size, writing condition, and sex.

Set Size	Stress Essay *M* (*SD*)	Neutral Essay *M* (*SD*)	Condition Difference *p*	Condition * Sex Interaction *p*
**2**	2.03 (0.99)	2.46 (0.92)	< .001	.268
**5**	0.98 (0.71)	1.15 (0.70)	.056	.750
**8**	0.63 (0.60)	0.77 (0.64)	.069	.881
**Total**	1.15 (0.63)	1.36 (0.61)	.006	.778

We next examined whether estimated parameters for attention, capacity, and guessing bias differed as a function of writing condition and sex. First, in a 2x2 ANOVA predicting attention from Condition and Sex a significant main effect of Condition emerged, *F*(1,273) = 9.11, *p* = .003, and a marginal Condition×Sex interaction, *F*(1, 273) = 3.64, *p* = .058, whereas the factor Sex was not a significant predictor, *p* = .598. Exploring the main effect of Condition in greater detail, we found that participants in the stressful writing condition (*M* = 0.866, *SE* = 0.008) had significantly lower attention to the task than participants in the neutral writing condition (*M* = 0.897, *SE* = 0.007). Next, in a 2x2 ANOVA predicting capacity from Condition and Sex, no significant main effect of Condition or sex and no significant Condition×Sex interaction emerged, *p*s>.104. Finally, in a 2x2 ANOVA predicting guessing bias from Condition and Sex, Sex emerged as a significant predictor, *F*(1, 273) = 13.90, *p* < .001, and a marginal main effect of condition emerged, *F*(1, 273) = 3.49, *p* = .063, whereas the Condition×Sex interaction was not significant, *p* = .451. Exploring Sex as a predictor in greater detail, we found that men (*M* = 0.625, *SE* = 0.013) showed a greater bias in guessing that a given square was “old” when than women (*M* = 0.561, *SE* = 0.011) (see Fig 4) Group means for each working memory outcome by writing condition and participant sex are reported in Table 2.

**Table 2:** Group means for each reported working memory outcome by participant condition and sex.

Working memory parameter	Stressful Writing/Men *M* (*SE*)	Neutral Writing/Men *M* (*SE*)	Stressful Writing/Women *M* (*SE*)	Neutral Writing/Women *M* (*SE*)
**Overall working memory**	1.16 (0.08)	1.39 (0.08)	1.14 (0.07)	1.33 (0.07)
**Attention**	0.85 (.01)	0.90 (0.01)	0.88 (.01)	0.89 (.01)
**Capacity**	2.40 (0.18)	2.57 (0.16)	2.22 (0.15)	2.58 (0.14)
**Guessing Bias**	0.60 (0.02)	0.65 (0.02)	0.55 (0.01)	0.57 (0.02)

**Fig 5:**
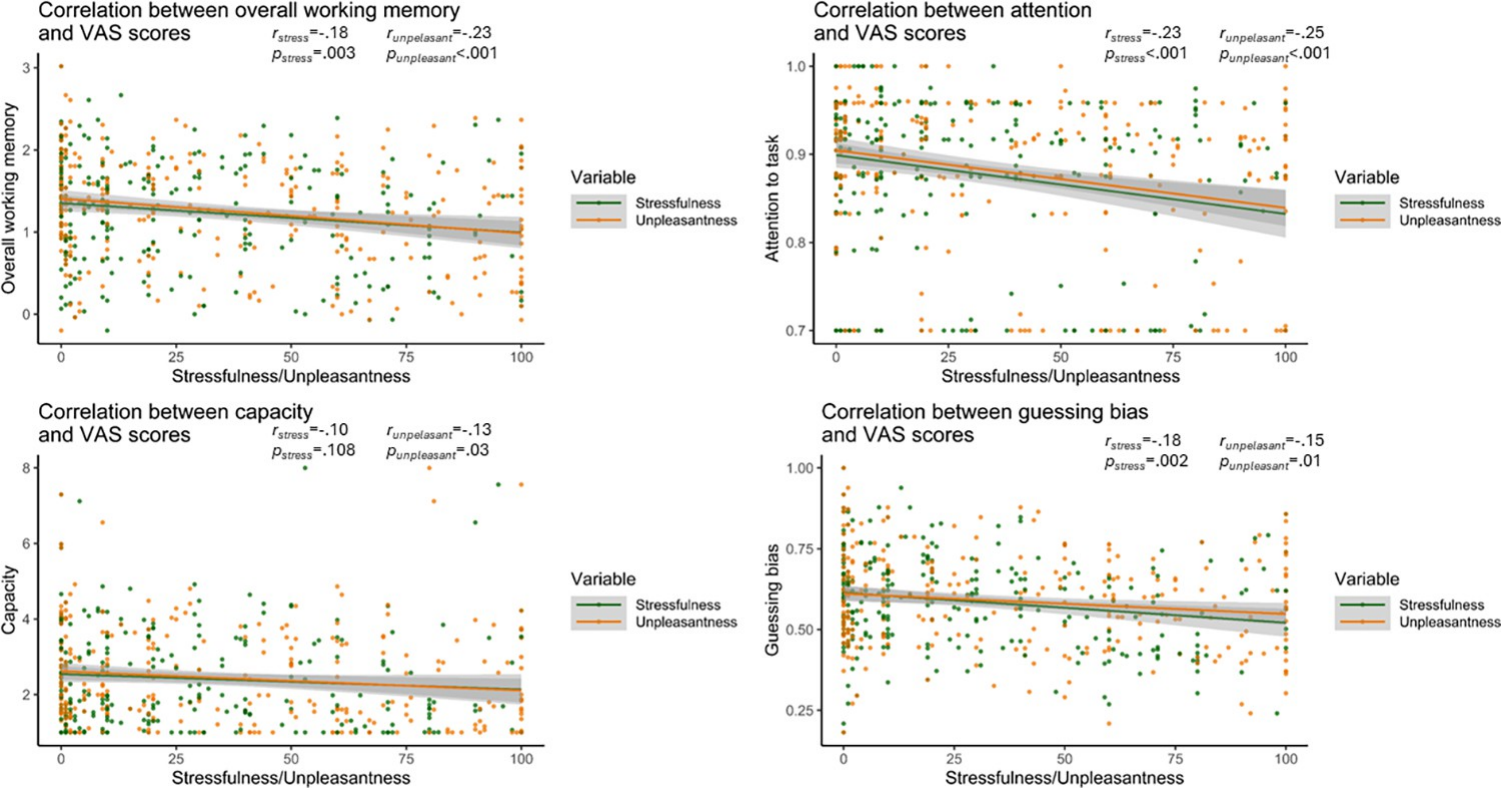
Correlations between overall working memory/working memory parameters and self-reported stressfulness/unpleasantness.

Additional analyses were examining bivariate correlations between self-reported stressfulness of the writing and working memory parameters, self-reported stressfulness of the task and working memory parameters, and overall essay sentiment and working memory parameters. There was a significant negative correlation between reported stressfulness of the task and overall working memory, such that greater reported stressfulness was associated with worse task performance, *r* = -.18, *p* = .003. Similarly, there was a significant negative correlation between reported stressfulness and attention to the task, such that greater stressfulness was associated with worse attention to the task, *r* = -.23, *p* < .001. There was also a significant negative correlation between stressfulness and guessing bias, such that greater stressfulness was associated with reduced bias in guessing that a square was “old”, *r* = -.18, *p* = .002. No significant associated was observed between stressfulness and capacity, *p* = .108. Similar results were obtained for reported unpleasantness of the task, with greater unpleasantness being significantly associated with reduced overall working memory, *r* = -.23, *p* < .001, reduced attention to the task, *r* = -.25, *p* < .001, and reduced bias in guessing that a square was “old”, *r* = -.15, *p* = .01. In contrast to stressfulness, a significant negative association was observed between unpleasantness and capacity, such that greater unpleasantness was associated with reduced capacity, *r* = -.13, *p* = .030 (see Fig 5).

Additionally, given the length of the change detection task, the effects of the writing manipulation may diminish over time. To address this possibility, we conducted additionally analyses examining working memory as a function of experimental condition and sex in the first and second half of test trials. In Task Half #1, participants in the stress essay condition (*M* = 1.25, *SE* = 0.06) showed worse working memory performance than participants in the control condition (*M* = 1.51, *SE* = 0.06), *t*(277) = 2.86, *p* = .005, Cohen’s *d* = 0.34. There was no Condition * Sex interaction in Task Half #1 working memory performance, *F*(1, 275) = 0.128, *p* = .721. Similarly, in Task Half #2, participants in the stress essay condition (*M* = 1.22, *SE* = 0.07) showed worse working memory performance than participants in the control condition (*M* = 1.48, *SE* = 0.06), *t*(277) = 2.75, *p* = .006, Cohen’s *d* = 0.33. There was no Condition * Sex interaction in Task Half #2 working memory performance, *F*(1, 275) = 0.107, *p* = .743.

Finally, we examined bivariate correlations between essay sentiment and working memory variables. No significant correlations were observed between overall essay sentiment and overall working memory, capacity, attention, or guessing bias, *p*s>.065.

## Discussion

Although stress influences working memory, the influences of thinking and writing about a prior but unresolved stressor on working memory is less clear. Results obtained throughout previous studies investigating the effects of writing about an unresolved stressful experience on cognitive functions suggested a previously unobserved interaction between stress and sex may be influencing working memory. We addressed this possibility by randomly assigning participants to either a stressful or neutral writing task, and subsequently assessing their working memory via a change detection task. Our results showed that writing about a stressful experience significantly reduced both overall working memory and attention, but that sex did not interaction with writing condition. Additionally, we found that across conditions stressfulness and unpleasantness of the writing task were significantly associated with poorer estimated attention within a working memory model.

The current study observed a significant main effect of writing condition, with participants that were assigned to write about an unresolved stressful experience showing an overall reduction in working memory and a reduction in attention to the task. Furthermore, across conditions we found that self-reported stressfulness and unpleasantness of the writing task were predictive of reductions in working memory. These findings lend considerable weight to the notion that writing about experiences that one deems stressful or unpleasant mimics experiencing an unpleasant stressful event. Consistent with theories of stress and cognition, it seems the writing task may impair working memory by diverting cognitive resources towards stressor relevant processes [2], with the degree of perceived unpleasantness of the writing task being predictive of greater impairment. These results are contrary to what would be expected from the mood-as-information theory of cognition and negative affect, which suggests that writing about a stressful experience and the increased negative affect that comes with should shift cognitive functions towards a more analytic style rather than impairing cognition [5]. This finding thus suggests that some writing-based negative affect inductions, such as stress or anxiety inductions [9], are more similar to an acute stressor than others. Future research should therefore continue to explore the impact writing-based stress inductions may have on other cognitive processes typically effected by acute stress, as well as the effect of other similar negative affect inductions on working memory.

The effects of the writing-based stress induction used in the current study notably also differ from the effects of other negative affect inductions on working memory. Negative affect induced via viewing negatively valenced pictures has been found to enhance visual working memory precision [16], and olfactory-induced negative affect inductions have been found to impair verbal working memory [17]. Similarly, at a meta-analytic level, negative affect inductions reduce working memory variability, attributed to increases in focal attention under negative arousal [18]. In sharp contrast, the present study observed an overall impairment in working memory performance and an impairment in focal attention. This contrast in findings highlights potential differences in the mechanisms underlying various means of affect induction as well as differences in the effects of inducing various affective states. Writing-based affect inductions involve a substantial cognitive component, possibly contributing to a shift in resource allocation towards the material being written about (i.e., the unresolved stressor). Writing-based inductions of anxiety have been found to impair cognitive flexibility [9], potentially suggesting a unique interplay between induced negative effect, the cognitive processes involved in writing-based manipulations, and executive functions.

In contrast to the obtained results, writing about stressful events has been studied as an intervention for stress and anxiety connected to unresolved past trauma [11, 12, 19–21]. This work has found that repeated instances of journaling interventions, during which individuals writing about past unresolved stressful experiences, result in greater improvements in mental and emotional health [11, 12, 19]. These psychological and emotional benefits can be attributed to an interplay between emotional and cognitive processing of the experience. Although this explanation suggests cognitive mechanisms play a role in the emotional benefits of writing, our current findings suggest an acute cognitive impairment. This finding is further complicated by previous work by the authors, in which we found that writing about an unresolved stressful event enhanced semantic clustering in an episodic memory task specifically in men in a free recall task [3]. Notably, these findings came from the same methods as the present study, just with a different outcome. Should the observed impairment in working memory be due to a shift in cognitive resources towards dealing with the unresolved stressor, this acute cognitive impairment should ultimately be predictive of long-term emotional benefits.

The current study hypothesized a potential writing condition by sex interaction that was not observed. Of note, though, we observed a significant main effect of condition, whereas the previous study conducted by Shields et al. [4] did not. There are a number of possible explanations for this difference in results. First, the present study’s sample was approximately 1.6 times larger than the previous one. As a result of this larger sample size, the present study had greater power to detect potential effects. It is possible that the current study merely detected the main effect of writing condition because of its greater power, especially given how relatively weak writing-based manipulations can be. However, this is unlikely, given the Bayesian results of Shields et al. [4]. An alternative explanation for these findings could be differences in experimental design, as we describe below.

Although the present study was designed to be a replication and extension of Shields et al. [4] the two studies differed in a few key ways. First, the present study was conducted online rather than in a laboratory setting. As a result, the location of a participant’s unresolved stressor may have been congruent with their location when completing the study. Additionally, environmental distractions could not be directly controlled by the researchers. Given context-dependent stress effects [22, 23], it is possible that this resulted in the two studies observing different effects of stressful writing on working memory. Additionally, the present study was part of a larger study that included a free recall task, with encoding taking place prior to the writing task and retrieval taking place immediately before the change detection task. Given potential time-dependent effects of stress on cognition [2, 24, 25] these differences in timing between prior work and the current study may explain the discrepant results observed. A delay between stress onset has been found to be predictive of greater working memory impairments at a meta-analytic level [2], therefore it may be the case that an aversive essay manipulation exerts time-dependent effects as well. Similarly, the change detection task used in the previous study contained a 500ms mask in which the previous locations were shown but covered in grey squares, whereas the present study contained no such mask. This mask was included to abolish traces of iconic memory (i.e., the fast-decaying traces of visual information held in sensory memory [26] and ensure that any observed effects could be attributed solely to working memory processes. Given that cortisol can decay iconic memory traces [14], it is possible that the results obtained in the present study are due to stress-related differences in iconic memory rather than working memory Future research should therefore attempt to test for this by distinctly examining the effects of writing about an unresolved stressor on both working memory and iconic memory.

Although this study has a number of strengths, including a large sample size, use of a well-validated working memory task, and comparability with prior work via the use of a previously developed and validated writing manipulation, there are a number of limitations that should be noted. First, data collection was conducted online during the height of the Covid-19 pandemic. Research conducted online during this period generally obtained weaker effect sizes than lab studies outside of the pandemic [27], but this potential issue has not been found to impact study generalizability [27]. Additionally, the Covid-19 pandemic impacted mental health, stress resilience, and immune responses [28–30]. To account for this, participant essays across conditions were examined for inclusion of Covid-related words (i.e., Covid, sick, cough, sneeze, congest, and breath). Working memory performance did not significantly differ between participants’ whose essays contained Covid-related words and those whose essays did not, *p* = .935. Similarly, because data were collected online, no physiological measures of stress (i.e., salivary cortisol) were assessed. Stressfulness was only measured via self-report, potentially impacting the true stressfulness of writing-based manipulations. Furthermore, the study we were seeking to replicate assessed baseline affect, whereas the current study did not. Participants baseline levels of negative affect may influence their writing content and task performance. Future research should therefore assess baseline affect prior to the writing task and physiological measures of stress following writing about an unresolved stressful experience. These results therefore may not generalize to participants with a difference in baseline stress. Third, the change detection task did not include a mask between stimulus onset and test. As a result, visual afterimages from task stimuli may have exerted an effect on participant performance rather than solely visual working memory [31]. Additionally, it is possible that, relative to passive waiting, the neutral writing task may have been stressful. We attempted to control for confounds related to typing and task type with our neutral writing task, but such a task may have enhanced working memory relative to waiting. Although this possibility would not explain differences between this study and the prior [4], future work could address this concern by using a control condition that does not involve writing. Finally, this study used a sample of college students recruited from psychology courses at a large public research university. As a result, the sample was considered Western, educated, industrialized, rich, and democratic, impacting generalizability of our results to non-Western cultures [32]. Future work should therefore attempt to replicate the present study outside of the Covid-19 pandemic and with a non-WEIRD sample to ensure generalizability of findings.

### Conclusion

In summary, we examined the effects of a single instance of writing about an unresolved stressful experience on working memory performance in a large sample of undergraduate participants and used computational cognitive modeling to further examine individual working memory processes. Our results showed that writing about a stressful experience, relative to writing about a neutral one, significantly reduced overall working memory performance and specifically reduced attention to the task. These results are consistent with theories of stress and cognition but contrary to what is expected from the mood-as-information theory of negative affect and cognition, suggesting that writing-based stress inductions have more similarities to acute stress inductions than with other negative affect inductions, especially when iconic memory positively contributes to working memory performance.
